# A surveillance method to identify patients with sepsis from electronic health records in Hong Kong: a single centre retrospective study

**DOI:** 10.1186/s12879-020-05330-x

**Published:** 2020-09-07

**Authors:** Ying Zhi Liu, Raymond Chu, Anna Lee, Charles David Gomersall, Lin Zhang, Tony Gin, Matthew T. V. Chan, William K. K. Wu, Lowell Ling

**Affiliations:** 1 Department of Anaesthesia and Intensive Care, The Chinese University of Hong Kong, Shatin, Hong Kong China; 2 Department of Anaesthesia and Intensive Care, Prince of Wales Hospital, Shatin, Hong Kong China

**Keywords:** Infection, Incidence, Sepsis, Population, Electronic health record, Asia

## Abstract

**Background:**

Currently there are only two population studies on sepsis incidence in Asia. The burden of sepsis in Hong Kong is unknown. We developed a sepsis surveillance method to estimate sepsis incidence from a population electronic health record (EHR) in Hong Kong using objective clinical data. The study objective was to assess our method’s performance in identifying sepsis using a retrospective cohort. We compared its accuracy to administrative sepsis surveillance methods such as Angus’ and Martin’s methods.

**Method:**

In this single centre retrospective study we applied our sepsis surveillance method on adult patients admitted to a tertiary hospital in Hong Kong. Two clinicians independently reviewed the clinical notes to determine which patients had sepsis. Performance was assessed by sensitivity, specificity, positive predictive value, negative predictive value and area under the curve (AUC) of Angus’, Martin’s and our surveillance methods using clinical review as “gold standard.”

**Results:**

Between January 1 and February 28, 2018, our sepsis surveillance method identified 1352 adult patients hospitalised with suspected infection. We found that 38.9% (95%CI 36.3–41.5) of these patients had sepsis. Using a 490 patient validation cohort, two clinicians had good agreement with weighted kappa of 0.75 (95% CI 0.69–0.81) before coming to consensus on diagnosis of uncomplicated infection or sepsis for all patients. Our method had sensitivity 0.93 (95%CI 0.89–0.96), specificity 0.86 (95%CI 0.82–0.90) and an AUC 0.90 (95%CI 0.87–0.92) when validated against clinician review. In contrast, Angus’ and Martin’s methods had AUCs 0.56 (95%CI 0.53–0.58) and 0.56 (95%CI 0.52–0.59), respectively.

**Conclusions:**

A sepsis surveillance method based on objective data from a population EHR in Hong Kong was more accurate than administrative methods. It may be used to estimate sepsis population incidence and outcomes in Hong Kong.

**Trial registration:**

This study was retrospectively registered at clinicaltrials.gov on October 3, 2019 (NCT04114214).

## Background

Global burden of severe sepsis is estimated to affect 48.9 million patients and accounts for 11 million deaths each year [1]. These latest estimates are significantly higher than previously reported figure of 19.4 million patients with sepsis globally each year [2]. This is because previous estimates were exclusively based on data from 26 high income western countries alone. The only exception was a single study from Taiwan, which provided an estimate based on a 1% sample of the population cohort [3]. Nevertheless, the updated estimate of sepsis incidence in 2020 is likely still lower than the real world incidence since it is computed from death registry data [1].

Sepsis incidence is often estimated from administrative sepsis surveillance methods such as Angus’ and Martin’s definitions [4–7]. These methods use readily available discharge coding and administrative data to estimate burden of sepsis. However variation in case definition of sepsis by diagnostic coding lead to very different incidence estimates of sepsis [8, 9]. Moreover, change in discharge coding habits result in an apparent but misleading change in incidence of sepsis over time [10]. Epidemiological research based on claims data alone is prone to bias as hospitals are reimbursed and paid accordingly. In addition, coding practice for sepsis has changed even in countries where coding is not used for insurance claims [11]. Increased coding for sepsis is in part due to better sepsis awareness rather than increasing true sepsis incidence alone [12, 13]. More recently, clinical data from electronic health records (EHR) have been used to estimate sepsis incidence [14, 15]. Compared to claims data, clinical data provides objective estimate that has more stable sepsis case definition and incidence over time [16].

There is currently limited epidemiological data on sepsis in Asia. Incidence of sepsis in Asia has mostly been exclusively studied in the Intensive Care Unit (ICU) [17–19]. Apart from a Taiwan population study, the only other population-based study from Asia is from a subdistrict of Beijing [3, 20]. Although Hong Kong is one of the most densely populated regions in Asia, the local estimate of sepsis incidence is unknown.

The purpose of this feasibility study is to develop an objective sepsis surveillance method based on a population EHR in Hong Kong to study incidence and outcomes of sepsis in this region. We evaluated its performance in identifying sepsis and compare its accuracy to Angus’ and Martin’s sepsis surveillance methods using a retrospective cohort of patients in Hong Kong.

## Method

### Study design

This was a single centre, retrospective study on hospitalised adult patients with infection or sepsis between January 1, 2018 and February 28, 2018. Patients were identified using the Clinical Data Analysis and Reporting System (CDARS), a population EHR database managed by the Hospital Authority, which provides more than 90% of acute care in Hong Kong [21, 22]. CDARS contains diagnoses, procedure codes, admission and discharge data, medication records, operation records, laboratory and microbiology results of all outpatient and inpatients treated in Hong Kong public hospitals since 1995. We applied our database sepsis surveillance method (explained below) to all patients admitted to a tertiary teaching hospital in Hong Kong during the study period. We validated our method against clinician’s manual review of clinical records and compared it to Angus’ and Martin’s definitions using the same cohort [4, 5]. This study was approved by The Joint Chinese University of Hong Kong – New Territories East Cluster Clinical Research Ethics Committee with waiver of informed consent (2019.214) and is registered on clinicaltrials.gov (NCT04114214). This manuscript was prepared using the STROBE guidelines for reporting observational studies.

### Identification of patients with suspected infection

**Fig. 1:**
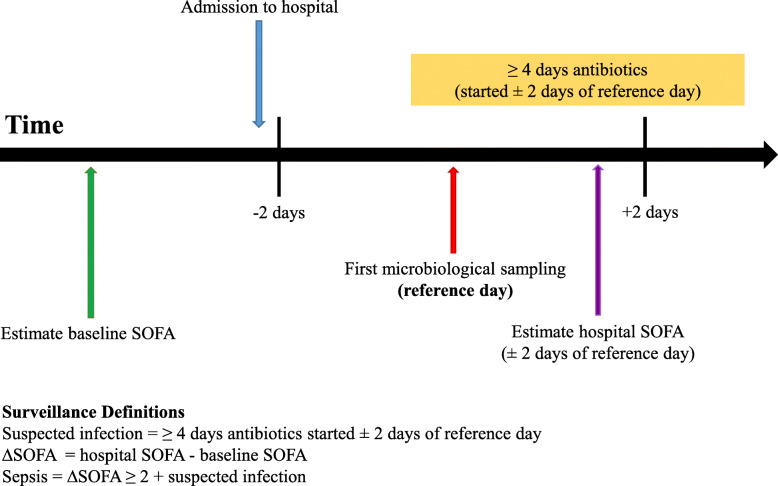
Sepsis Surveillance Method. Schematic diagram on use of objective clinical data from different time points according to date of first microbiological sampling as reference day

To identify patients with suspected infection within CDARS, we first identified all inpatients at who had at least one bacterial sensitivity or culture test during the study period (Fig. 1). We excluded patients who only had screening microbiological tests such as methicillin-resistant *Staphylococcus aureus* nasal swab or vancomycin-resistant *Enterococci* rectal swab as these were performed for cohort screening rather than on suspicion of infection. First microbiological sampling date was set as reference day for each patient. Criteria on use of antibiotics to identify patients with infection are based on the United States Centre for Disease Control Prevention Hospital Toolkit for Adult Sepsis Surveillance (ASE) [23]. Patients were excluded if antibiotics were not started 2 days before or after first microbiological sampling. They were also excluded if duration of antibiotics was less than 4 days unless death occurred before the fourth day. Lastly, patients discharged without antibiotics before the fourth day were also excluded.

### Identification of patients with sepsis

To identify patients with sepsis we applied the Sepsis 3 definition to patients identified with suspected infection from CDARS [24, 25]. Patients with suspected infection with an estimated increase in sequential organ failure assessment (SOFA) score of ≥2 were classified as patients with sepsis. Patients with suspected infection but estimated ∆SOFA < 2 were classified as uncomplicated infection. The estimated ∆SOFA score is calculated by the difference between hospital SOFA and pre-hospital (baseline) SOFA. Similar to previous studies, hospital discharge diagnostic/procedural coding and clinical data were used to estimate each component of SOFA (supplementary Table 1) [14, 15]. Hospital SOFA is the estimated SOFA within 2 days of first microbiological sampling. Baseline SOFA is defined as the estimated SOFA score before hospitalisation. For hospital SOFA estimation, only respiratory and neurological SOFA component included discharge diagnosis coding. All other organ scores were based on objective clinical data from laboratory results or procedure codes. Patients who did not have at least one of the three lab results (bilirubin, platelet or creatinine) measured within 2 days of first microbiological sampling were excluded. Consistent with previous methods, all missing values for components of SOFA were assumed to be normal [14, 15]. Lastly we calculated the proportion of patients with missing laboratory data to assess for bias.

### Validation

Similar to previous studies, we used a clinician reviewed validation cohort to assess the performance of our sepsis surveillance method [14, 15]. Based on data from the United States, we assumed a sepsis prevalence of 50% amongst hospitalised patients with infection, expecting a sensitivity or specificity of 0.99, lower confidence limit of 0.04, a significance level of 0.05 and power of 0.95, the minimum sample size for a validation cohort was 208 in each group using “MKmisc” [14, 26]. Anticipating missing data and diagnoses, we used a computer to randomly select 245 patients with sepsis and 245 patients with uncomplicated infection as classified by our surveillance method for clinician review. Two clinicians, blinded to the surveillance method results, each reviewed the clinical notes and laboratory results to determine whether patients had infection or sepsis according to Sepsis-3 definition [25]. Clinician disagreements were resolved by discussion. Sensitivity, specificity, positive predictive value (PPV), negative predictive value (NPV), positive likelihood ratio (PLR), negative likelihood ratio (NLR) and area under the curve (AUC) of the receiver operating characteristic curve were used to assess the performance of our sepsis surveillance method, Angus’ and Martin’s definitions compared to classification by clinician review [4, 5].

### Statistical analyses

Normality of continuous data was assessed by visual inspection and Shapiro-Wilk’s test. The chi-square test or Fisher exact test were used to compare differences in proportion. For non-parametric comparisons, Wilcoxon rank sum test was used and median with interquartile range (IQR) were reported. Cohen’s kappa coefficient test was used to measure the inter-observer agreement between clinicians. Statistical analysis were performed in SPSS (Version 22.0) and RStudio (Version 3.6). Level of significance was set at *p* < 0.05.

## Results

### Cohort identification and characteristics

**Fig. 2:**
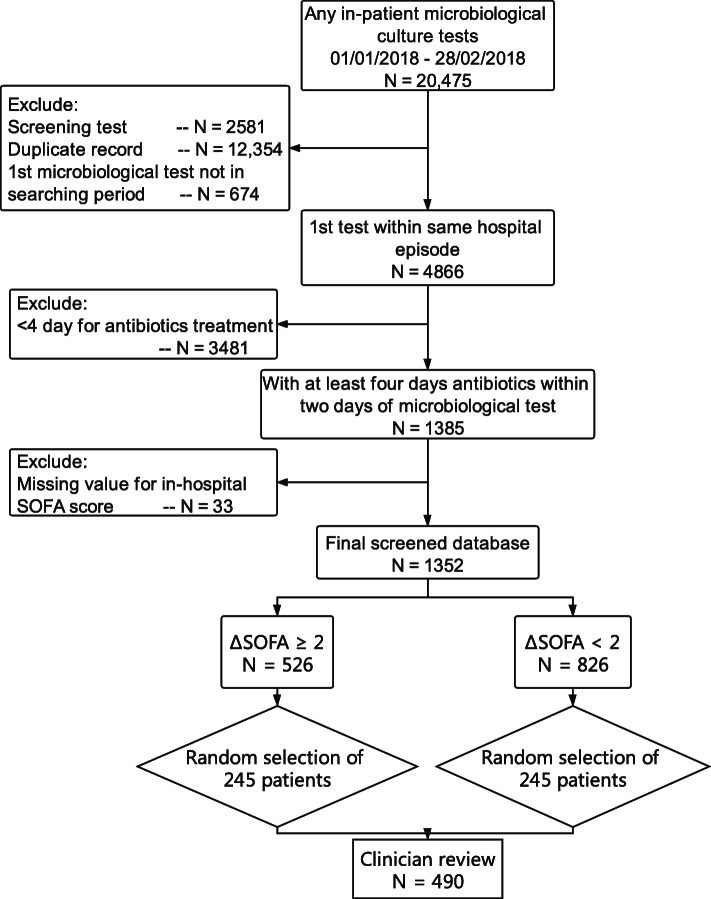
Cohort identification. Flow chart showing identification of patients from a population electronic health database who were admitted to a tertiary hospital in Hong Kong

From January 1, 2018 to February 28, 2018, there were 20,475 inpatient microbiological culture tests performed at Prince of Wales Hospital in Hong Kong (Fig. 2). After exclusions, there were 1385 unique adult patients who had antibiotics initiated within 2 days of first microbiological sampling and prescribed continuously for more than 4 days. Within this group, 33 patients were excluded because they did not have any laboratory results for in-hospital SOFA scoring. A final cohort of 1352 patients with suspected infection was identified. Of these 826 (61.1, 95%CI 58.5–63.7%) had uncomplicated infection whilst 526 (38.9, 95%CI 36.3–41.5%) had a ∆SOFA score of ≥2 and thus were classified as having sepsis. There were 3 patients in the uncomplicated infection group who had ∆SOFA = − 1 because their hospital SOFA was less than baseline SOFA.

The baseline characteristics of 490 patients in the validation cohort are shown in Table 1. The 28-day mortality for patients with sepsis in our cohort was 50/245 (20.4, 95%CI 15.7–25.8). The source of infection in patients were 41.6% (204/490) respiratory, 13.9% (68/490) gastrointestinal, 13.3% (65/490) urinary, 10.2% (50/490) unknown, 5.9% (29/490) musculoskeletal, 2.4% (12/490) skin, 1.6% (8/490) primary bacteraemia, 1.2% (6/490) nervous system, 0.8% (4/490) ear nose throat, 0.6% (3/490) gynecological/obstetrics and 9.4% (46/490) not infection. There were 3 patients with multiple sources of infection. The most common antibiotics used were 71.0% (348/490) co-amoxiclav, 32.7% (160/490) tazobactam/piperacillin and 13.5% (66/490) ceftriaxone. Blood cultures were performed in 17.9% (40/224) of patients identified with sepsis by clinician review. Proportion of patients with missing laboratory data are shown in supplementary Table S3. Of patients with missing baseline laboratory results, their median (IQR) hospital bilirubin was 13.8 (9.1–21.3) μmol/L, platelet 191 (137–251) × 10^3^/μL and creatinine 87 (67–117) μmol/L.

**Table 1:** Baseline characteristics and outcomes of validation cohort

	Infection	Sepsis	*P* Value
	N = 245	*N* = 245	
Age, years, median (IQR)	69.7 (58.6–83.4)	77.10 (64.0–85.1)	0.007
Age group, No. (%)			0.013
< 60	68 (27.8)	42 (17.1)	
60–69	55 (22.4)	48 (19.6)	
70–79	38 (15.5)	53 (21.6)	
≥ 80	84 (34.3)	102 (41.6)	
Male sex, No. (%)	124 (50.6)	145 (59.2)	0.057
Community acquired infection^*^, No. (%)	223 (91.0)	206 (84.1)	0.02
Positive culture, No. (%)	109 (44.5)	106 (43.3)	0.785
Charlson Comorbidity Index (Non Age Adjusted), median (IQR)	1.0 (0.0–2.00)	1.0 (0.0–3.0)	0.068
Charlson Comorbidity Index group, No. (%)			0.211
0	116 (47.3)	93 (38.0)	
1	35 (14.3)	43 (17.6)	
2	39 (15.9)	47 (19.2)	
≥ 3	55 (22.4)	62 (25.3)	
Estimated Baseline SOFA, median (IQR)	0 (0–1)	0 (0–1)	0.113
Estimated Hospital SOFA, median (IQR)	0 (0–1)	4 (2–6)	< 0.001
Number of organ dysfunction, No. (%)			< 0.001
0	129 (52.7)	0 (0.0)	
1	96 (39.2)	58 (23.7)	
2	19 (7.8)	105 (42.9)	
3	1 (0.4)	53 (21.6)	
≥ 4	0 (0.0)	29 (11.8)	
Respiratory Dysfunction, No. (%)			
Baseline	0 (0.0)	0 (0.0)	–
New/Worsening	3 (1.2)	48 (19.6)	< 0.001
Hospital	3 (1.2)	48 (19.6)	< 0.001
Neurological Dysfunction, No. (%)			
Baseline	28 (11.4)	21 (8.6)	0.292
New/Worsening	10 (4.1)	57 (23.3)	< 0.001
Hospital	38 (15.5)	78 (31.8)	< 0.001
Cardiovascular Dysfunction, No. (%)			
Baseline	0 (0.0)	0 (0.0)	–
New/Worsening	0 (0.0)	32 (13.1)	< 0.001
Hospital	0 (0.0)	32 (13.1)	< 0.001
Liver Dysfunction, No. (%)			
Baseline	0 (0.0)	9 (3.7)	0.01
New/Worsening, No. (%)	12 (4.9)	103 (42.0)	< 0.001
Hospital	12 (4.9)	105 (42.9)	< 0.001
Hematological Dysfunction, No. (%)			
Baseline	8 (3.3)	27 (11.0)	0.001
New/Worsening	30 (12.2)	131 (53.5)	< 0.001
Hospital	32 (13.1)	139 (56.7)	< 0.001
Renal Dysfunction, No. (%)			
Baseline	36 (14.7)	38 (15.5)	0.280
New/Worsening	27 (11.0)	124 (50.6)	< 0.001
Hospital	52 (21.2)	145 (59.2)	< 0.001
Emergency surgery, No. (%)	31 (12.7)	33 (13.5)	0.789
ICU admission, No. (%)	1 (0.4)	33 (13.5)	< 0.001
Hospital length of stay, days, median (IQR)	9.0 (5.0–18.0)	13.0 (7.0–23.0)	< 0.001
28-day mortality, No. (%)	12 (4.9)	50 (20.4)	< 0.001

^*^Positive bacterial cultures within 48 h of hospital admission

Dysfunction for each system is any estimated SOFA component ≥1. If patient had higher estimated SOFA component in hospital than their baseline score it is considered new/worsening dysfunction

*ICU* intensive care unit, *IQR* interquartile range

### Performance of our sepsis surveillance method

Clinicians had an initial inter-rater agreement of weighted kappa of 0.75 (95% CI 0.69–0.81). They were able to reach consensus on diagnosis of uncomplicated infection or sepsis for all cases after discussion. The performance of our method, Angus’ and Martin’s methods are shown in Table 2. Compared to clinician review our method misidentified sepsis in 53 patients (supplementary Table S2). The most common reason was due to non-infection (15/53). Other reasons for incorrect identification were due to inaccurate or missing objective data: dementia code (8/53), oxygen therapy code (9/53), non-invasive ventilation code (7/53), baseline creatinine (3/53), baseline platelet (6/53), baseline bilirubin (2/53), hospital GCS score (1/53), long term home oxygen therapy code (1/53) and mechanical ventilation without respiratory failure (1/53).

**Table 2:** Sepsis surveillance performance of different methods

	Our Method	Angus [4]	Martin [5]
**Sensitivity**	0.93 (95%CI 0.89–0.96)	0.12 (95%CI 0.08–0.17)	0.19 (95%CI 0.14–0.25)
**Specificity**	0.86 (95%CI 0.82–0.90)	0.99 (95%CI 0.97–1.00)	0.92 (95%CI 0.88–0.95)
**PPV**	0.85 (95% CI 0.81–0.88)	0.90 (95%CI 0.74–0.98)	0.66 (95%CI 0.55–0.78)
**NPV**	0.93 (95%CI 0.90–0.96)	0.57 (95%CI 0.53–0.62)	0.57 (95%CI 0.53–0.62)
**PLR**	6.68 (95%CI 4.94 to 9.02)	10.69 (95%CI 3.29 to 34.76)	2.32 (95%CI 1.43 to 3.76)
**NLR**	0.08 (95%CI 0.05 to 0.13)	0.89 (95%CI 0.85 to 0.94)	0.88 (95%CI 0.82 to 0.95)
**AUC**	0.90 (95% CI 0.87–0.92)	0.56 (95%CI 0.53–0.58)	0.56 (95%CI 0.52–0.59)

*AUC* area under the curve, *PLR* positive likelihood ratio, *PPV* positive predictive value, *NLR* negative likelihood ratio, *NPV* negative predictive value

## Discussion

We developed a sepsis surveillance method based on stored objective clinical data from a population EHR in Hong Kong. It had an AUC of 0.90 (95% CI 0.87–0.92) to distinguish patients with sepsis from a cohort of hospitalised adult patients with suspected infection. It performed better than Angus’ and Martin’s sepsis surveillance methods in capturing patients with sepsis from EHR in Hong Kong. Since coding system and laboratory results are shared across all public hospitals in Hong Kong, it may be feasible to use our surveillance method to calculate the local population incidence of sepsis in this region.

In our cohort, we found that 39% of patients hospitalised with infection developed sepsis, which is similar to data from the United States [14]. Similar to studies that included non-ICU patients, we showed that 86.5% of septic patients in this cohort were managed outside the ICU [27, 28]. Therefore sepsis epidemiological studies that only capture septic patients in the ICU would underestimate the burden of sepsis. This is particularly important in Hong Kong and other parts of Asia since critical care resources are limited compared to western countries [29, 30]. Overall, 28-day mortality for all patients with sepsis in our cohort was 20.4% (95%CI 15.7–25.8), which is lower than the hospital mortality of 44.5% (95%CI 41.8–47.2) for patients with sepsis described in the multicentre MOSAICS study from Asian ICUs [31]. The lower overall estimate of sepsis mortality is because our method captured both patients with less severe sepsis treated on the wards and patients with severe sepsis who are managed in the ICU.

Our approach differs from previously published objective methods to estimate sepsis from EHR in several ways. First previous sepsis surveillance methods either did not account for preadmission comorbidities or assumed best laboratory values during hospitalisation as baseline values [4, 14]. In contrast, we accounted for patient’s pre-hospital organ dysfunction to generate a baseline organ function score and is similar to a recently reported sepsis surveillance method from Valik et al. in Sweden [15]. Thus we captured patients who at baseline had organ dysfunction and were admitted to hospital with an infection but did not constitute sepsis since there were no changes in their organ dysfunctions from baseline. Epidemiologically it is important to distinguish patients with sepsis from patients who have infection with a background of organ dysfunction as their pathophysiology differ. Second, similar to Valik et al.’s method, we used graded SOFA component scores to account for severity of organ dysfunction [15, 25]. This is different to threshold based scoring where each organ either has or has not failed [14]. Third, we identified cases of suspected infection by any microbiological sampling rather than blood culture alone since the latter assumes septic patients always have blood cultures performed. In fact, in our cohort 82.1% of septic patients identified by clinician review did not have blood cultures taken. Thus using Rhee’s et al.’s approach of defining onset of sepsis by blood culture is not feasible in our setting [14]. Lastly, we used the US CDC’s ASE definition of 4 days of antibiotics rather than 2 doses that Valik *et. al* used. Together, these differences explain why our method appears to be more sensitive but less specific compared to Rhee et al.’s (sensitivity 0.697 and specificity 0.981) and Valik et al.’s (sensitivity 0.887 and specificity 0.985) methods [14, 15].

Sepsis surveillance using administrative and coding data have generally shown a steady increase in sepsis incidence over time with decreasing mortality [5, 32]. However estimates from clinical data showed minimal change in population incidence of sepsis [14]. Locally, our study demonstrated that coding based sepsis surveillance such as the Angus’ or Martin’s methods have very low sensitivity to identify septic patients in Hong Kong. This would result in significant underestimation of sepsis burden in Hong Kong. This is likely due to incomplete discharge summary coding in our population EHR. Likewise, low sensitivity but high specificity of these two diagnostic coding based methods have been reported in patient cohorts based in the United States [33].

In contrast, several studies have demonstrated the utility of using objective clinical data from EHR to provide consistent sepsis surveillance [14, 15]. This has particular advantages as it is less prone to deficiencies in coding, change in coding practices or bias from claims benefits. However there is a need validate this approach in diverse populations and different EHR systems [34]. Our study is the first to demonstrate that this a feasible approach in an Asian healthcare setting and population to provide accurate and consistent sepsis epidemiological data. In Asia, the National Electronic Health Record of Singapore, Hospital Information of Thailand and Hospital Information System of West China are large EHR that contain laboratory test values, procedure records and diagnosis of more than 11 million patients combined [35]. Apart from Hong Kong, it may be possible to apply a similar sepsis surveillance method in these countries and regions with existing EHR databases to provide much needed local population estimates of sepsis.

There are a few limitations with our sepsis surveillance method. First, oxygen and non-invasive ventilation use were not captured well as it was often not recorded in the EHR. Second, we used first microbiological sampling as reference day which identified community acquired sepsis better than for hospital acquired sepsis. We could not capture patients who had cultures taken despite initial admission for non-infectious reasons and then subsequently developed nosocomial sepsis. These limitations are not unique to our method and are also weaknesses in previous studies [16]. Third, our method falsely attributed some non-infectious causes of multi-organ failure as sepsis. Fourth, we are unable to capture hypotension treated with fluid boluses since regular blood pressures recordings are not available. Fifth, we had to use either a single GCS reading upon hospital or ICU admission or diagnostic coding due to missing data. Sixth, we assumed patients had normal values for missing laboratory tests, which is an accepted limitation in previous methods [14, 15]. Although 22.7–27% of patients did not have prehospital bilirubin, platelet or creatinine results, these patients had normal median hospital bilirubin, platelet and creatinine. Normal hospital laboratory results suggests these were likely previously healthy patients with normal baseline results. It supports the assumption for baseline SOFA component score of 0 in patients with missing baseline data. In addition, the low Charlson Comorbidity Index in our cohort is in agreement with these assumptions. Furthermore, only 0.2–2.2% of patients in our validation cohort had missing bilirubin, platelet or creatinine results. Seventh, it cannot identify septic patients in whom infection is not recognised by the treating clinicians and thus no microbiological samples were sent or no antibiotics were given. Lastly we are unable to identify patients who were not admitted to hospital but treated for sepsis in the community. As this was a single centre feasibility study, further multicentre data would be needed to validate our surveillance method in Hong Kong.

## Conclusion

In this feasibility study we found that an objective sepsis surveillance method based on clinical data from a population EHR in Hong Kong to identify sepsis was more accurate than methods based on administrative data. Although burden of sepsis in Hong Kong is currently unknown, our surveillance method may be used to provide an accurate estimate of population incidence and outcomes of sepsis in Hong Kong.

## Supplementary information

**Additional file 1.** Supplementary Table S1. Table S1 Calculation of Baseline and Hospital SOFA. Diagnosis and procedural codes are in ICD-9-CM. ^a^Patients on long term oxygen or have dementia before hospitalisation have both prehospital and hospital SOFA of 2 and thus would not affect ∆SOFA. ^b^Patients who are on renal replacement therapy before hospitalisation have both prehospital and hospital SOFA of 4 and thus would not affect ∆SOFA. Abbreviation: GCS, Glasgow coma scale; SOFA, sequential organ failure assessment

**Additional file 2.** Supplementary Table S2. Table S2 Clinician review compared to sepsis surveillance methods. Diagnosis of uncomplicated infection or sepsis in 490 patient cohort according to clinician review compared to sepsis surveillance methods.

**Additional file 3.** Supplementary Table S3. Table S3 Missing Laboratory Values. Proportion of patients in validation cohort with missing laboratory values.

## Data Availability

The datasets used and/or analysed during the current study are available from the corresponding author on reasonable request. Approval from The Joint Chinese University of Hong Kong – New Territories East Cluster Clinical Research Ethics Committee will be required before sharing of data.

## Abbreviations

AUC: area under the curve
CDARS: Clinical Data Analysis and Reporting System
EHR: electronic health records
GCS: Glasgow Coma Scale
ICU: Intensive Care Unit
IQR: interquartile range
NPV: negative predictive value
PPV: positive predictive value
SOFA: sequential organ failure assessment
