# Supplementary material for: A surveillance method to identify patients with sepsis from electronic health records in Hong Kong: a single centre retrospective study
Source: BMC Infect Dis. 2020 Sep 7;20:652. doi: 10.1186/s12879-020-05330-x (PMC7487694; doi:10.1186/s12879-020-05330-x)
Supplement: Supplementary file 1 — Additional file 1. Supplementary Table S1. Table S1 Calculation of Baseline and Hospital SOFA. Diagnosis and procedural codes are in ICD-9-CM. aPatients on long term oxygen or have dementia before hospitalisation have both prehospital and hospital SOFA of 2 and thus would not affect ∆SOFA. bPatients who are on renal replacement therapy before hospitalisation have both prehospital and hospital SOFA of 4 and thus would not affect ∆SOFA. Abbreviation: GCS, Glasgow coma scale; SOFA, sequential organ failure assessment [file 12879_2020_5330_MOESM1_ESM.docx]

**Table S1 Calculation of Baseline and Hospital SOFA**

| **Prehospital (Baseline) SOFA** | | |
| --- | --- | --- |
|  | **Score** | **Conditions** |
| **Respiratory** | 0 | All patients assumed to be zero at baseline |
|  | 2^*^ | Diagnostic code of V46.2 supplemental oxygen or Clinical Data Framework Long Term oxygen) |
|  | | |
| **Neurological** | 0 | All patients assumed to be zero at baseline |
|  | 2^*^ | Diagnostic code of 290 dementias, 294 persistent mental disorders due to conditions classified elsewhere, 317 – 319 mental retardation, 331.0 Alzheimer’s disease, 331.1 frontotemporal dementia, 331.2 senile degeneration of brain, 331.82 dementia with lewy bodies, 331.83 mild cognitive impairment |
|  | | |
| **Cardiovascular** | 0 | All patients assumed to be zero at baseline |
|  | | |
| **Hepatic**  **(Bilirubin, μmol/L)**  (lowest bilirubin within 1 year to 1 week before admission) | 0 | <20 |
|  | 1 | 20−32 |
|  | 2 | 33−101 |
|  | 3 | 102−204 |
|  | 4 | >205 |
|  | | |
| **Hematological**  **Platelet (10^3^/µL)**  (highest platelet within 1 year to 1 week before admission) | 0 | ≥150 |
|  | 1 | 101−149 |
|  | 2 | 51−100 |
|  | 3 | 21−50 |
|  | 4 | ≤20 |
|  |  |  |
| **Renal**  **Creatinine (μmol/L)** (lowest creatinine within 1 year to 1 week before admission) | 0 | ≤109 |
|  | 1 | 110−170 |
|  | 2 | 171−299 |
|  | 3 | 300−440 |
|  | 4 | >440  or  Procedure code of 39.95 hemodialysis, 54.98 peritoneal dialysis, V56.0 renal dialysis encounter^†^ |
|  | | |
| **Hospital SOFA** | | |
|  | **Score** | **Conditions** |
| **Respiratory**  **(P_a_O_2_/F_i_O_2_, mmHg)**  (lowest verified result) or diagnostic/procedural code | 0 | >400  or  Lack of verified P_a_O_2_/F_i_O_2_ and data |
|  | 1 | 301−400  or  Diagnostic code of 518.81 respiratory failure, 518.82 other pulmonary insufficiency |
|  | 2 | 201−300  or  Procedure code of 96.04 non-invasive mechanical ventilation, 96.7 continuous invasive mechanical ventilation of unspecified duration, 96.71 continuous invasive mechanical ventilation for less than 96 consecutive hours, 96.72 continuous invasive mechanical ventilation for 96 consecutive hours or more or 96.7 other continuous invasive mechanical ventilation |
|  | 3 | 101−200 |
|  | 4 | ≤100 |
|  | | |
| **Neurological** | 0 | GCS 15 |
|  | 1 | GCS 13−14  or  Diagnostic code of 293.0 or 293.1 delirium, 348.31 encephalopathy, 780.97 altered mental status |
|  | 2 ^*^ | GCS 10−12  or  Diagnostic code of 290 dementias, 294 persistent mental disorders due to conditions classified elsewhere, 317 – 319 mental retardation, 331.0 Alzheimer’s disease, 331.1 frontotemporal dementia, 331.2 senile degeneration of brain, 331.82 dementia with lewy bodies, 331.83 mild cognitive impairment |
|  | 3 | GCS 6−9 |
|  | 4 | GCS <6 |
|  | | |
| **Cardiovascular** | 0 | All patients assumed to be zero unless there is diagnostic coding |
|  | 1 | Diagnostic code of 458 hypotension |
|  | 2 | Procedural code of  00.17 infusion of vasopressor agent  or  use of dopamine, dobutamine, noradrenaline, adrenaline, vasopressin within 2 days of microbiological sampling |
|  | | |
| **Hepatic**  **(Bilirubin, μmol/L)** (highest bilirubin within 2 days of first microbiological sampling) | 0 | <20 |
|  | 1 | 20−32 |
|  | 2 | 33−101 |
|  | 3 | 102−204 |
|  | 4 | >205 |
|  | | |
| **Hematological**  **Platelet (10^3^/µL)** (lowest platelet within 2 days of first microbiological sampling) | 0 | ≥150 |
|  | 1 | 101−149 |
|  | 2 | 51−100 |
|  | 3 | 21−50 |
|  | 4 | ≤20 |
|  | | |
| **Renal**  **Creatinine (μmol/L)** (highest creatinine within 2 days of first microbiological sampling) | 0 | ≤109 |
|  | 1 | 110−170 |
|  | 2 | 171−299 |
|  | 3 | 300−440 |
|  | 4 | >440  or  Procedure code of 39.95 hemodialysis, 54.98 peritoneal dialysis, 38.95 venous catheterisation for renal dialysis, V56.0 renal dialysis encounter |

Diagnosis and procedural codes are in ICD-9-CM.

^*^Patients on long term oxygen or have dementia before hospitalisation have both prehospital and hospital SOFA of 2 and thus would not affect ∆SOFA.

^†^Patients who are on renal replacement therapy before hospitalisation have both prehospital and hospital SOFA of 4 and thus would not affect ∆SOFA.

GCS, Glasgow Coma Scale; SOFA, sequential organ failure assessment
