# Supplementary material for: A surveillance method to identify patients with sepsis from electronic health records in Hong Kong: a single centre retrospective study
Source: BMC Infect Dis. 2020 Sep 7;20:652. doi: 10.1186/s12879-020-05330-x (PMC7487694; doi:10.1186/s12879-020-05330-x)
Supplement: Supplementary file 2 — Additional file 2. Supplementary Table S2. Table S2 Clinician review compared to sepsis surveillance methods. Diagnosis of uncomplicated infection or sepsis in 490 patient cohort according to clinician review compared to sepsis surveillance methods. [file 12879_2020_5330_MOESM2_ESM.docx]

**Supplementary Table S2 Clinician review compared to sepsis surveillance methods**

|  | | **Clinician review** | |
| --- | --- | --- | --- |
|  |  | **Sepsis** | **Infection** |
| **Our method** | **Sepsis** | 208 | 37 |
|  | **Infection** | 16 | 229 |
| **Angus’ method** | **Sepsis** | 27 | 3 |
|  | **Infection** | 197 | 263 |
| **Martin’s method** | **Sepsis** | 43 | 22 |
|  | **Infection** | 181 | 244 |
