# Supplementary material for: A surveillance method to identify patients with sepsis from electronic health records in Hong Kong: a single centre retrospective study
Source: BMC Infect Dis. 2020 Sep 7;20:652. doi: 10.1186/s12879-020-05330-x (PMC7487694; doi:10.1186/s12879-020-05330-x)
Supplement: Supplementary file 3 — Additional file 3. Supplementary Table S3. Table S3 Missing Laboratory Values. Proportion of patients in validation cohort with missing laboratory values. [file 12879_2020_5330_MOESM3_ESM.docx]

**Supplementary Table S3 Missing Laboratory Values**

|  | **Missing Data (%)** |
| --- | --- |
| **Baseline Bilirubin** | 135/490 (27.6) |
| **Baseline Platelet** | 134/490 (27.3) |
| **Baseline Creatinine** | 111/490 (22.7) |
| **Hospital Bilirubin** | 11/490 (2.2) |
| **Hospital Platelet** | 1/490 (0.2) |
| **Hospital Creatinine** | 5/490 (1.0) |
